# Supplementary material for: Chromothripsis during telomere crisis is independent of NHEJ, and consistent with a replicative origin
Source: Genome Res. 2019 May;29(5):737–49. doi: 10.1101/gr.240705.118 (PMC6499312; doi:10.1101/gr.240705.118)
Supplement: Supplemental Material [file supp_gr.240705.118_Supplemental_file_1.zip › contigs/annotated_contigs/DB53/contig.3.DB53_length_570_mean_cov_14.049122807.docx]

**DB53_length_570_mean_cov_14.049122807**

```
ATGAGGTACCT|TGGTTTTTTGTTGGTTTTTCTTTTTTT|TTTTTTTTTTTTTTTGAGATGGAGTCTCGCTCTGTTGCCCAGGCTGGAG
            >chr1:48792823-48792850 - E=1e-01 p=1e-03  >chr5:31816257-31816409 - E=9e-7
TGCAGTGGCGCGATCTCGGCTCACTGCAAGCTCCGCCTCCTGGGTTCATGCTATTCTCCTGCCTCAGCCTCCCGAGTAGCTGGGACTAC
1
AGGCGCCTGCCACC|TG|TATATATATACACATACTTGGCTTGGGACTAAAAAAACCAAGGCATTGAGAAAAGAAAAAGTAACTCATGA
                 >chr12:23753477-23753855 + E=7e-216
CAATATGTCAGGCATTAGTGCAAACAGTTCACAAACAGTAAGTTCAAGGACCTGTAACTCAACAAGCAACAATATGCATGTTCTTGAAT

GCAGATTTTGAACAGACCAGATTTCTTTTTGTGTTTTCACATTTACAGAACACAACTCCAGTGTGCCGTTTTCAGAGGTTCACTGTGAG

TCCTCTAAAGGTTTCTTTATCATTTAATATTATAAATAATTATGCTCCTGGGCTGCAGTTCAAAGCTCAGTACAGCACTCGTATAACAC

CACAGGCAGGTCAGGTGACCTTTCTCCAATTCTCCTCTCT
```
